# Supplementary material for: Visualization of Runs of Homozygosity and Classification Using Convolutional Neural Networks
Source: Biology (Basel). 2025 Apr 16;14(4):426. doi: 10.3390/biology14040426 (PMC12025119; doi:10.3390/biology14040426)
Supplement: Supplementary file 1 [file biology-14-00426-s001.zip › File 4 Supplementary Materials.pdf]

Model Validation and Comparison

We implemented a CNN model with 10-fold cross-validation to train the neural network on image data. The dataset was partitioned into 10 subsets, with each subset serving sequentially as the validation set, while the remaining subsets were used for training. This approach provides a robust evaluation of model stability and helps prevent overfitting. For each fold, a model was created using create_model(), trained for 100 epochs (epochs = 100), and the training history and performance metrics were stored in the cv_results list.

For comparison with classical machine learning approaches, logistic regression was selected for binary image classification. Due to the high dimensionality of the image vectors (512 × 512 × 3 = 786,432 features), dimensionality reduction was first applied using Principal Component Analysis (PCA). The images were flattened into one-dimensional vectors, and PCA was performed using the robust SVD algorithm (RSpectra::svds). The logistic regression model was evaluated using 10-fold cross-validation (trainControl(method = "cv", number = 10)) with class balancing (upsampling strategy), and performance was assessed based on the ROC-AUC metric (twoClassSummary) implemented in the caret package.

A comparative visualization of the performance of both models is presented below.

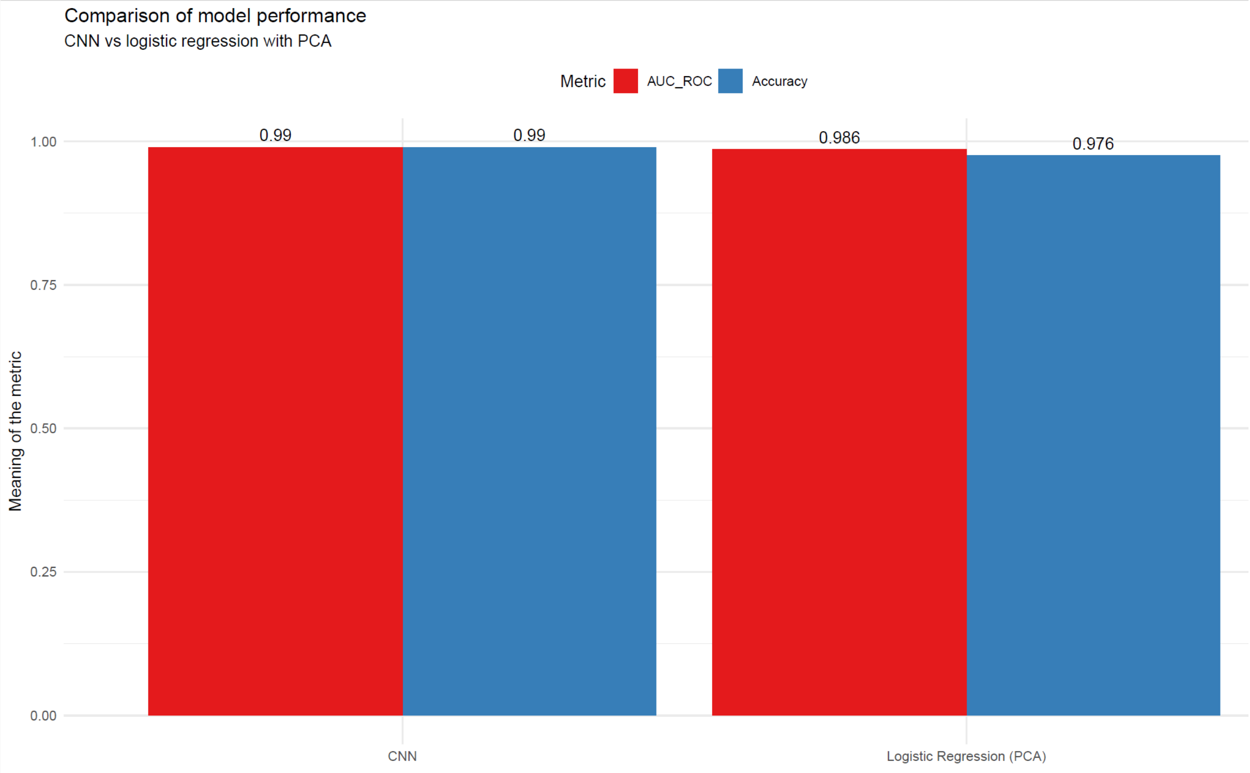

Figure S3. Visualization of the performance of both models

Comparative Limitations of Logistic Regression for Image Analysis

While logistic regression demonstrates acceptable performance metrics, it remains fundamentally ill-suited for image analysis in our context. Unlike CNNs, it fails to account for the spatial organization of image data. Even after dimensionality reduction via PCA, the model operates on global statistical

patterns while entirely disregarding local image features—such as textures, edges, and contours-that are essential for meaningful interpretation.

The linear architecture of logistic regression imposes critical limitations: its inability to model nonlinear pixel interactions significantly hampers the recognition of complex visual patterns compared to CNNs. Being inherently linear, it cannot capture the hierarchical feature representations and nonlinear dependencies that are crucial for identifying subtle variations within regions of homozygosity.

In contrast, CNNs naturally learn such representations through convolutional layers that preserve spatial relationships and adapt effectively to data variability. This architectural advantage underpins their superior performance in detecting visual patterns in ROH data, as they automatically extract multi-level feature hierarchies that logistic regression is fundamentally incapable of modeling.

We subsequently applied Saliency Maps (Kadir & Brady, 2001) to achieve fine-grained interpretation of our CNN's decision-making process. As a result, it enables the precise identification of specific pixel intensity changes that contribute most significantly to the classification decision, going beyond merely highlighting generally important image regions.

In our CNN-based analysis of genomic data, this approach proved especially valuable for accurately localizing homozygosity regions and identifying fine-scale patterns that influence classification. Saliency Maps reveal the most influential pixels (or genomic positions) affecting model outputs, capturing subtle but biologically meaningful local features that may be overlooked by Grad-CAM due to its reliance on aggregated higher-layer activations. This precision makes Saliency Maps particularly useful for assessing CNN reliability, as they can identify model vulnerabilities where minor input variations might lead to misclassification.

For all test data, Saliency Maps were used to determine the regions of highest importance-image areas most relevant for distinguishing homozygosity patterns between the two breeds. To facilitate comparison, we generated average saliency maps for each breed by first normalizing the maps to a 0–255 range and then applying binary thresholding (Simonyan et al., 2013) to retain only the most significant areas. To further improve the clarity of the maps, we applied morphological operations: opening (erosion followed by dilation) to remove isolated noise pixels, and closing (dilation followed by erosion) to fill gaps within contiguous regions (Gonzalez & Woods, 2018). These post-processing steps produced smoother and more coherent importance regions, which are essential for accurate localization in genomic image analysis, where even minor visual artifacts can affect interpretation.

The resulting visualizations include both representative examples from each breed (Figure S4) and the corresponding averaged saliency maps (Figure S5), highlighting the critical regions driving classification decisions.

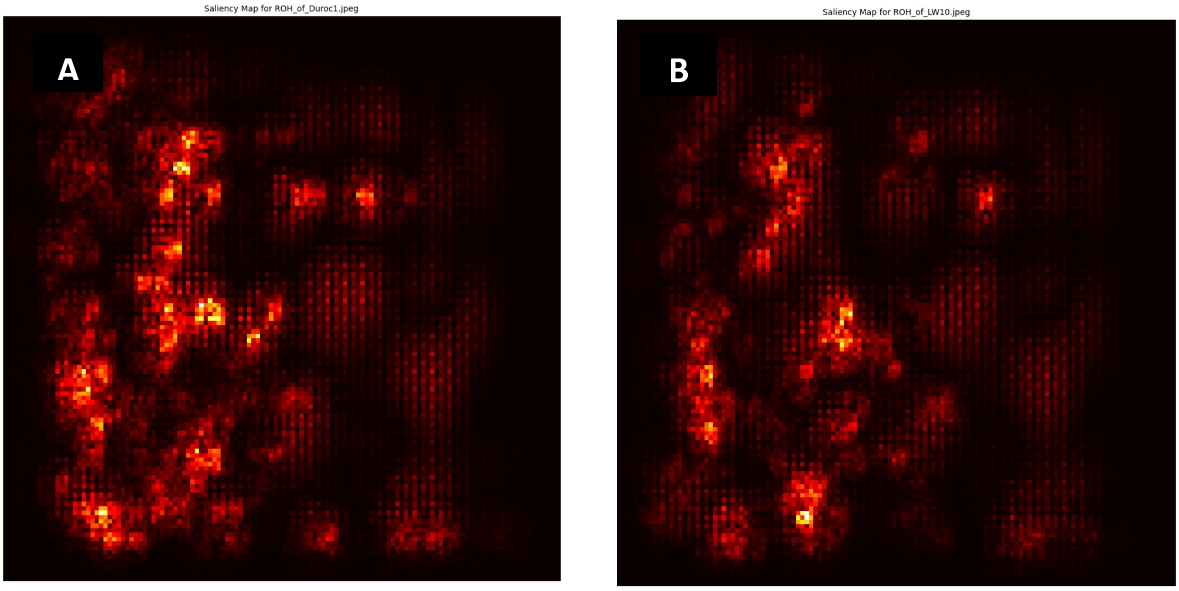

Figure S4. Importance regions for individuals of the Duroc (A) and Large White (B) breeds

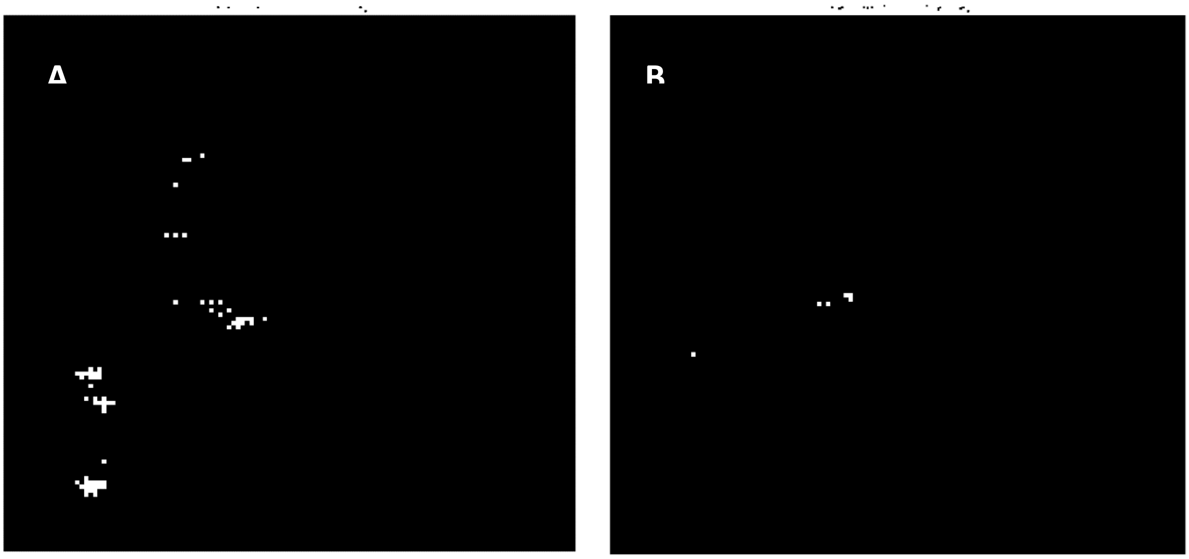

Figure S5. Average saliency maps for Duroc (A) and for Large White (B)
