# Supplementary material for: Visualization of Runs of Homozygosity and Classification Using Convolutional Neural Networks
Source: Biology (Basel). 2025 Apr 16;14(4):426. doi: 10.3390/biology14040426 (PMC12025119; doi:10.3390/biology14040426)
Supplement: Supplementary file 1 [file biology-14-00426-s001.zip › File 2 Supplementary Materials.pdf]

Table S1. Summary Statistics of ROH for Duroc and Large White

	Min	1st Qu	Median	Mean	3rd Qu	Max
Large White	1220	3451	4703	5523	6289	42415
Duroc	1225	3663	4957	6017	7050	48604

Table S2. The distribution of ROH by segment length for Duroc and Large White

Count	1-2	2-4	4-6	6-8	8-16	>16
Large White						
Count	540	4944	5158	2027	1895	298
Count (%)	3.633	33.266	34.706	13.639	12.751	2.005
Duroc						
Count	566	7630	8287	4139	4382	656
Count (%)	2.205	29.735	32.295	16.130	17.077	2.557

Table S3. Summary Statistics of ROH for Large White pigs based on the presence or absence of limb defects

	Min	1st Qu	Median	Mean	3rd Qu	Max
LW1	1002	2188	3247	4935	5586	76133
LW2	1003	2195	3254	5134	5907	68751

Table S4. The distribution of ROH by segment length for Large White pigs based on the presence or absence of limb defects

Count	1-2	2-4	4-6	6-8	8-16	>16
LW1						
Count	6318	12693	5143	2629	3038	1320
Count (%)	20.288	40.759	16.515	8.442	9.756	4.238
LW2						
Count	3547	7207	2808	1512	1992	847
Count (%)	19.801	40.233	15.676	8.441	11.120	4.728
