# Supplementary material for: Visualization of Runs of Homozygosity and Classification Using Convolutional Neural Networks
Source: Biology (Basel). 2025 Apr 16;14(4):426. doi: 10.3390/biology14040426 (PMC12025119; doi:10.3390/biology14040426)
Supplement: Supplementary file 1 [file biology-14-00426-s001.zip › File 3 Supplementary Materials.pdf]

Table S5. Confusion Matrix and Performance Metrics for Breed Classification

Confusion Matrix:

**Prediction**	**Reference: ROH_of_Duroc**	**Reference: ROH_of_LW**
ROH_of_Duroc	10	0
ROH_of_LW	0	10

Performance Metrics:

- **Accuracy:** 1 (100%)
  95% Confidence Interval: (0.8316, 1)
- **No Information Rate:** 0.5
- **P-Value [Acc > NIR]:** 9.537e-07
- **Kappa:** 1
- **Mcnemar's Test P-Value:** NA

Class-Specific Metrics:

- **Sensitivity (True Positive Rate):** 1.0
- **Specificity (True Negative Rate):** 1.0
- **Positive Predictive Value (Precision):** 1.0
- **Negative Predictive Value:** 1.0
- **Prevalence:** 0.5
- **Detection Rate:** 0.5
- **Detection Prevalence:** 0.5
- **Balanced Accuracy:** 1.

2. ROC Curve for Evaluating the Performance of Breed Classification

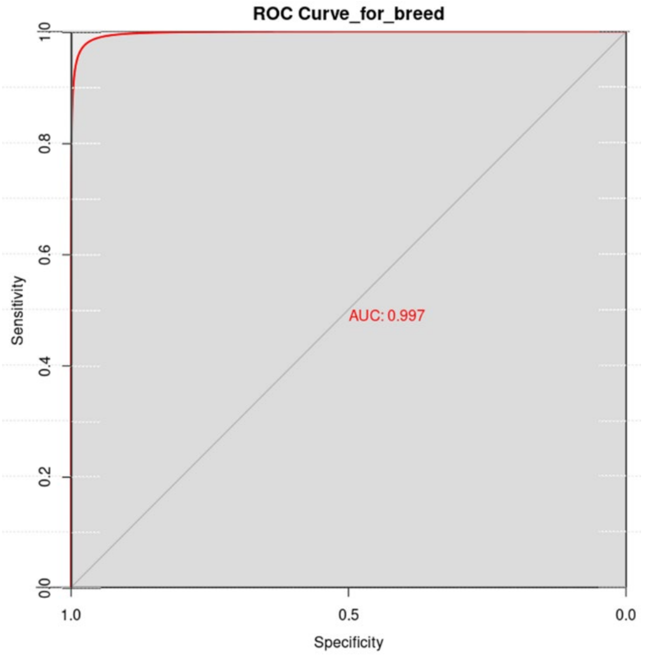

Figure S1. ROC curve for evaluating the performance of Breed Classification

(The area under the curve (AUC) is 0.997)

Table S6. Confusion Matrix and Performance Metrics for Leg Defect Classification

Confusion Matrix:

Prediction	Reference: 0 (No Defect)	Reference: 1 (Defect)
0 (No Defect)	22	4
1 (Defect)	5	11

Performance Metrics:

- **Accuracy:** 0.7857 (78.57%)
  95% Confidence Interval: (0.6319, 0.897)
- **No Information Rate:** 0.6429
- **P-Value [Acc > NIR]:** 0.03466
- **Kappa:** 0.5401

Class-Specific Metrics:

- **Sensitivity (True Positive Rate):** 0.7333 (73.33%)
- **Specificity (True Negative Rate):** 0.8148 (81.48%)
- **Positive Predictive Value (Precision):** 0.6875 (68.75%)
- **Negative Predictive Value:** 0.8462 (84.62%)
- **Prevalence:** 0.3571 (35.71%)
- **Detection Rate:** 0.2619 (26.19%)
- **Detection Prevalence:** 0.3810 (38.10%)
- **Balanced Accuracy:** 0.7741 (77.41%)

Note: The 'Positive' Class is defined as **1 (Defect)**.

4. ROC Curve for Evaluating the Performance of for Leg Defect Classification

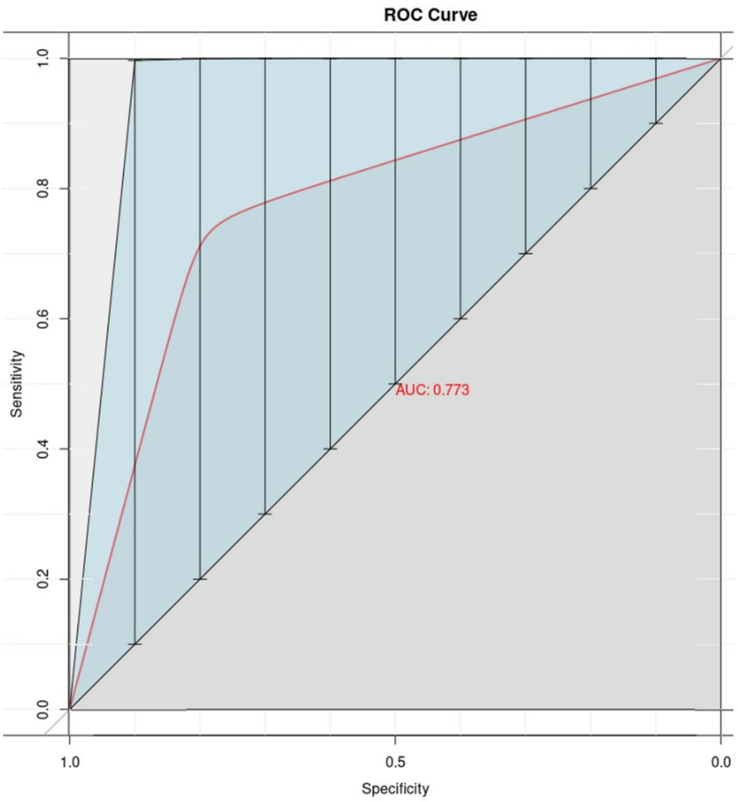

Figure S2. ROC curve for evaluating the performance of the Leg Defect classification

(The area under the curve (AUC) is 0.773)
